# NLRX1 knockdown attenuates pro-apoptotic signaling and cell death in pulmonary hyperoxic acute injury

**DOI:** 10.1038/s41598-023-28206-x

**Published:** 2023-03-01

**Authors:** Hye Rin Kim, Mi Na Kim, Eun Gyul Kim, Ji Su Leem, Seung Min Baek, Yu Jin Lee, Kyung Won Kim, Min-Jong Kang, Tae Won Song, Myung Hyun Sohn

**Affiliations:** 1 Department of Pediatrics, Severance Hospital, Institute of Allergy, Institute for Immunology and Immunological Diseases, Severance Biomedical Science Institute, Graduate School of Medical Science, Brain Korea 21 Project, Yonsei University College of Medicine, 50-1 Yonsei-ro, Seodaemun-gu, Seoul, 03722 Korea; 2 Section of Rheumatology, Allergy and Immunology, Department of Internal Medicine, Yale University School of Medicine, New Haven, CT USA; 3 Department of Pediatrics, Ilsan Paik Hospital, Inje University College of Medicine, 170 Juhwa-ro, Ilsanseo-gu, Goyang, 10380 Korea

**Keywords:** Immunology, Molecular medicine

## Abstract

Hyperoxia is frequently used for treating acute respiratory failure, but it can cause acute lung injury. Nucleotide-binding domain and leucine-rich-repeat-containing family member X1 (NLRX1) is localized in mitochondria and involved in production of reactive oxygen species, inflammation, and apoptosis, which are the features of hyperoxic acute lung injury (HALI). The contribution of NLRX1 to HALI has not previously been addressed. Thus, to investigate the role of NLRX1 in hyperoxia, we generated a murine model of HALI in wild-type (WT) and NLRX1^−/−^ mice by exposure to > 95% oxygen for 72 h. As a result, NLRX1 expression was elevated in mice exposed to hyperoxia. In acute lung injury, levels of inflammatory cells, protein leakage, cell cytotoxicity, and pro-inflammatory cytokines were diminished in NLRX1^−/−^ mice compared to WT mice. In a survival test, NLRX1^−/−^ mice showed reduced mortality under hyperoxic conditions, and apoptotic cell death and caspase expression and activity were also lower in NLRX1^−/−^ mice. Furthermore, levels of the MAPK signaling proteins ERK 1/2, JNK, and p38 were decreased in NLRX1-deficient mice than in WT mice exposed to hyperoxia. The study shows that a genetic deficit in NLRX1 can suppress hyperoxia-induced apoptosis, suggesting that NLRX1 acts as a pivotal regulator of HALI.

## Introduction

Oxygen is essential for human survival, and it is sometimes used as adjuvant therapy for patients with respiratory failure or as a means of increasing oxygen delivery to peripheral tissues in patients with severe lung or heart disease^1^. However, prolonged exposure to high concentrations of oxygen has been shown to increase DNA fragmentation and levels of reactive oxygen species (ROS) that induce apoptosis in pulmonary tissue^2^. Excessive accumulation of these free radicals leads to acute and chronic lung injury^2^.

Hyperoxia is defined as a supraphysiological concentration and pressure of oxygen in cells, tissues, or organs. When the lungs are exposed to high concentrations of oxygen, oxygen gradually replaces nitrogen as the primary gas in alveoli and places the lungs in a state of absorption atelectasis due to a reduction in lung volume, because of resorptive processes that occur with oxygen but not with nitrogen^3^. Hyperoxia increases production of oxidant species via the mitochondrial nicotinamide adenine dinucleotide phosphate oxidase pathway; hence, prolonged hyperoxia causes mitochondrial dysfunction^4,5^. Hyperoxic acute lung injury (HALI) is characterized by damage and death of endothelial and epithelial cells, which results in leakage of alveolar capillary proteins^1,6^. Continuous exposure to hyperoxia can contribute to the pathogenesis of various lung diseases, including chronic obstructive pulmonary disease (COPD), asthma, idiopathic pulmonary fibrosis, and acute respiratory distress syndrome (ARDS)^7^.

Nucleotide-binding domain and leucine-rich-repeat-containing family member X1 (NLRX1) is a protein expressed and localized in mitochondria^8,9^. Recent research has shown that NLRX1 has roles in modulation of mitochondrial function, generation of ROS, autophagy, and apoptosis^8,10–13^. The NLRX1 acts as a mitochondrial controller of apoptotic cell death in ischemia–reperfusion injury and acute cellular injury^10,14^. In addition, other studies showed that NLRX1 is involved in numerous diseases, including COPD, cancer, deafness, and tumorigenesis^13,15,16^. However, studies on NLRX1 signaling under hyperoxia have been lacking.

Mitogen-activated protein kinase (MAPK) signaling pathways, with ERK 1/2, JNK, and p38 subfamilies, mediate key cellular processes, including regulation of cell survival and death. MAPK pathways may act as either activators or inhibitors, depending on the cell type and the stimulus^17,18^. Studies of various pulmonary diseases, including respiratory failure, fibrosis, neutrophilic inflammatory disease, and HALI, have reported that MAPK pathways are activated in association with increased apoptosis^6,19–23^. In addition, it has been suggested that NLRX1 modulates MAPK pathways in immune responses to stimuli, including viral or fungal infection and tumorigenesis^24–27^.

In the present study, we aimed to identify the role of NLRX1 in HALI. We hypothesized that gene deletion of NLRX1 would have effects on lung damage and mouse survival under hyperoxic conditions. Using an NLRX1 null mutant, we identified the basic cellular and signaling features in acute lung injury, from inflammation to apoptosis and mortality, and determined signaling pathways active under hyperoxic conditions.

## Results

### Hyperoxia elevates NLRX1 expression, and NLRX1 deficiency attenuates hyperoxia-induced lung injury

**Figure 1:**
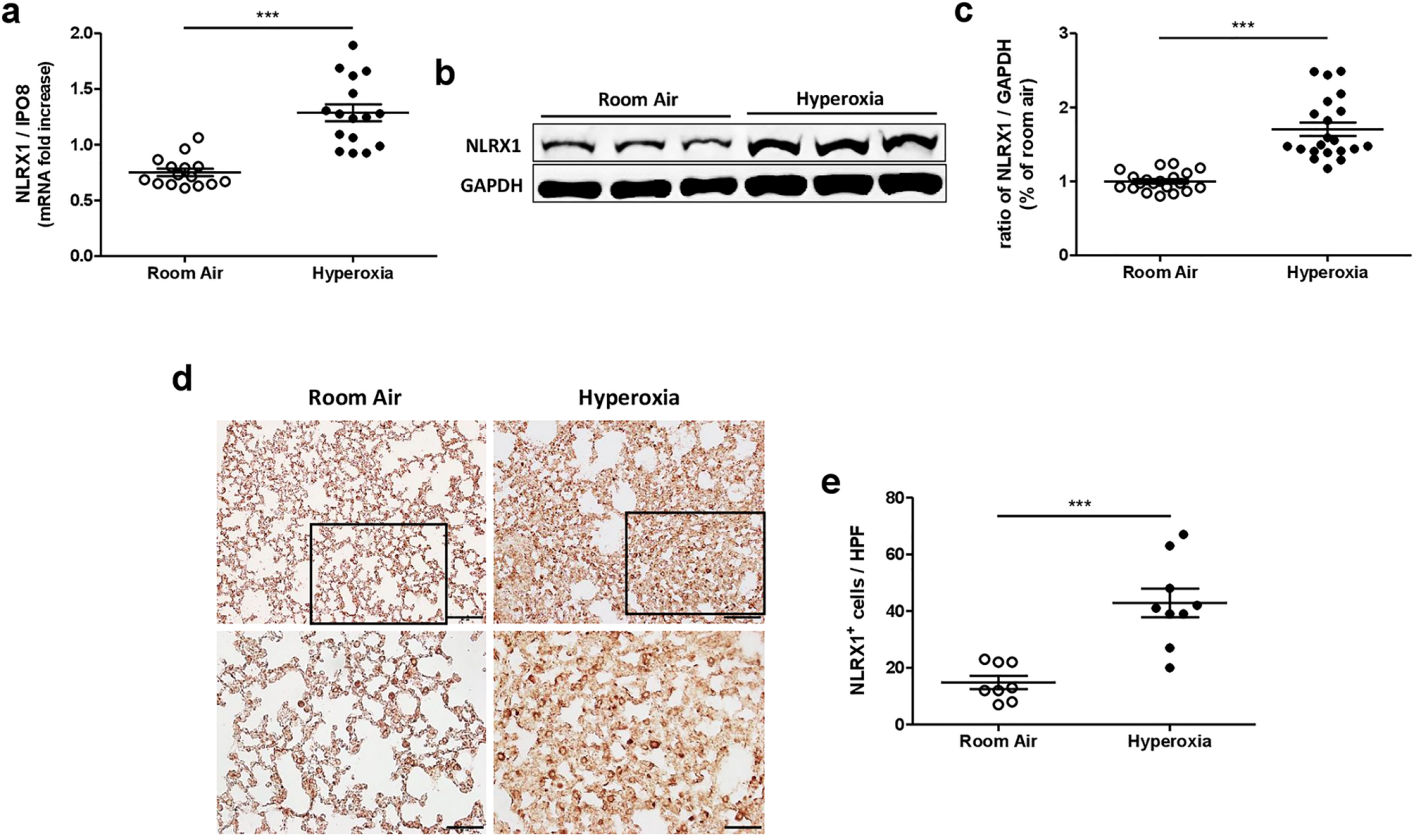
NLRX1 expression levels are increased in a murine model of hyperoxia. Wild-type mice were exposed to room air (n = 5) or hyperoxia (≥ 95% oxygen) (n = 5) respectively for 72 h. After 72 h, mice were sacrificed and collected lung tissues were analyzed. (**A**) mRNA levels for NLRX1 in lung lysates were measured by real-time PCR. (**B**) NLRX1 protein expression was evaluated via western blot using lung lysates, and (**C**) the signal intensity of NLRX1 was quantified. (**D**) Immunohistochemical staining of NLRX1 was assessed in mouse lung tissue, and (**E**) NLRX1 positive cells were counted. Scale bars 100 µm and 50 µm. Results are presented as the mean ± SEM and are representative of at least three independent experiments. The images of the original blots are available in Supplementary Information. Some blots were cut prior to hybridization with antibodies. ***p < 0.001 (Student’s *t* test). *HPF* high power field.

To determine whether NLRX1 is modulated by hyperoxia, we established an experimental murine model of hyperoxia and analyzed the expression of NLRX1. Wild-type (WT) mice were exposed to either hyperoxic air (HO, > 95% oxygen) or room air (RA) for 72 h. NLRX1 mRNA and protein levels were higher in hyperoxia-treated mice than in control mice (Fig. 1A–C). NLRX1 immunohistochemical staining in lung tissue also confirmed that NLRX1 levels are elevated following hyperoxia (Fig. 1D,E).

**Figure 2:**
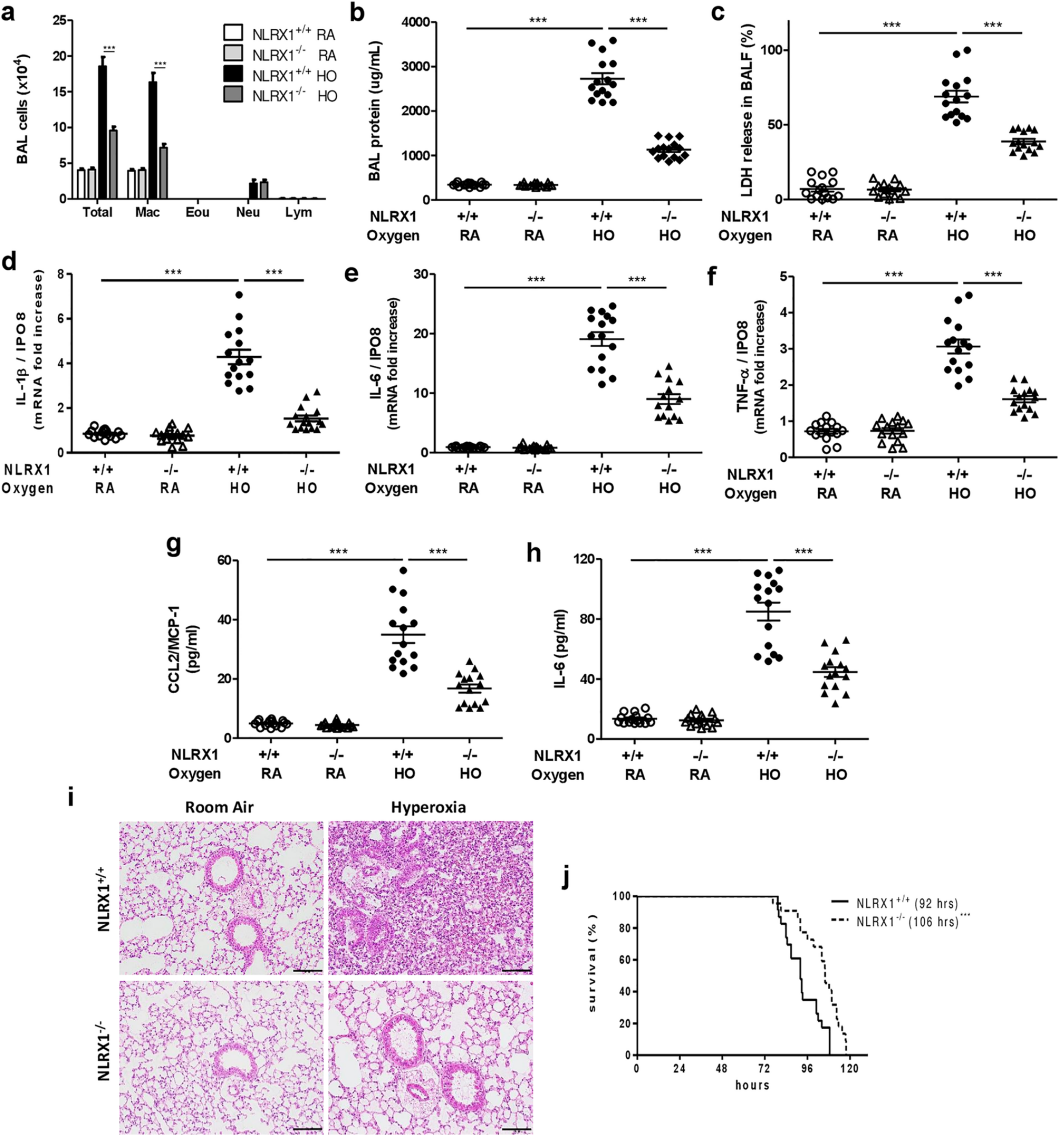
NLRX1 deficiency dampens the inflammation and mortality induced by hyperoxia. Wild-type mice (NLRX1^+/+^) and NLRX1 knock-out mice (NLRX1^−/−^) were exposed to both room air (RA) and hyperoxia (HO, ≥ 95% oxygen) for 72 h. (**A**) Inflammatory cells from bronchoalveolar lavage (BAL) fluid were counted. (**B**) Total protein concentration of BAL fluid was quantified by BCA assay. (**C**) Cell cytotoxicity in BAL fluid was assessed using LDH assay. (**D**–**F**) mRNA levels of inflammatory cytokines IL-1β, IL-6, and TNF-α were examined using real-time PCR in lung tissues. (**G**,**H**) CCL2 and IL-6 levels were analyzed by ELISA in BAL fluid. Results are presented as the mean ± SEM and are representative of at least three independent experiments (n = 5 mice per group). (**I**) Lung tissues were histologically analyzed for lung injury and inflammation by optical microscope after staining with hematoxylin and eosin (H&E). Scale bars 100 µm. (**J**) Mortality rates in WT and NLRX1^−/−^ mice were measured by survival test (NLRX1^+/+^; n = 23, NLRX1^−/−^; n = 22) and corrected for multiple comparisons by the log-rank test. The median survival (LD_50_) is in parentheses. Data represent at least three separate experiments. ***p < 0.001 (two-way ANOVA and Student’s *t* test).

Since hyperoxia increased NLRX1 levels, we compared the severity of acute lung injury between WT and NLRX1^−/−^ mice. Numbers of inflammatory cells, total protein concentrations, and cellular cytotoxicity in bronchoalveolar lavage (BAL) fluid were increased in WT mice following hyperoxia, but NLRX1^−/−^ mice showed reduced inflammatory responses after hyperoxic exposure, as compared to WT mice (Fig. 2A–C).

To determine whether NLRX1 contributes to hyperoxia-induced proinflammatory cytokine expression, we examined mRNA and protein levels of proinflammatory cytokines IL-1β, IL-6, TNF-α, and CCL2, using real-time polymerase chain reaction (PCR) and enzyme-linked immunosorbent assay (ELISA), respectively. Increased mRNA and protein levels for these cytokines were detected in lung tissues and BAL fluid in hyperoxia-treated mice, but NLRX1^−/−^ mice showed reduced inflammatory responses compared to WT mice (Fig. 2D–H). Hematoxylin–eosin staining of lung tissue revealed lower levels of inflammation in NLRX1^−/−^ mice than that in WT mice (Fig. 2I).

To evaluate mortality, the end point of acute lung injury and survival was measured in WT and NLRX1^−/−^ mice under prolonged hyperoxia exposure. NLRX1^−/−^ mice had significantly longer survival times than did WT mice (median survival; LD_50_ was 106 h in NLRX1^−/−^ and 92 h in WT mice) (Fig. 2J). The data confirm that hyperoxia-induced acute lung injury is significantly less severe in NLRX1^−/−^ mice.

### NLRX1 knockout reduces hyperoxia-induced apoptosis

**Figure 3:**
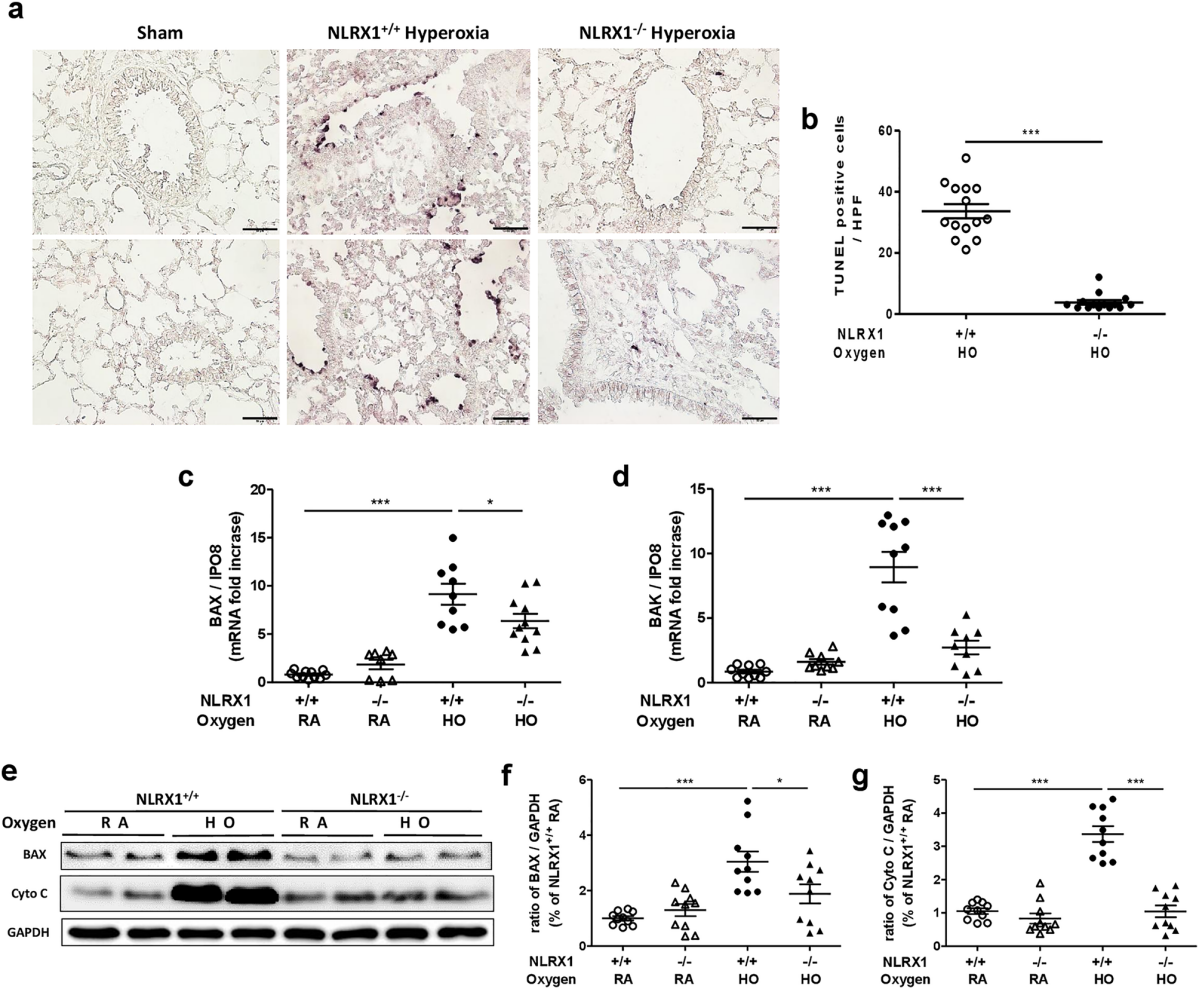
Absence of NLRX1 decreases the number of apoptotic cells and signaling molecules under hyperoxic conditions. (**A**) NLRX1^+/+^ room air (sham), NLRX1^+/+^ hyperoxia, and NLRX1^−/−^ hyperoxia mice lung sections were stained using TUNEL assay and (**B**) TUNEL-positive cells were counted. Scale bars 50 µm. (**C**,**D**) mRNA levels of *Bax* and *Bak* were measured by real-time PCR. () Protein levels of BAX and Cytochrome C (Cyto C) were analyzed using western blot of lung lysates, and (**F**,**G**) the ratio of molecules was calculated. Results are presented as the mean ± SEM and are representative of at least three independent experiments (n = 5 mice per group for real-time PCR and n = 2 mice per group for western blot). The images of the original blots are available in Supplementary Information. Some blots were cut prior to hybridization with antibodies. *p < 0.05, ***p < 0.001 (Student’s *t* test).

Following the observation of the difference in survival after NLRX1 knockout, we used a terminal deoxynucleotidyl transferase (TdT)-mediated dUTP nick end labeling (TUNEL) assay to detect DNA fragmentation and determine whether NLRX1 modulates hyperoxic apoptosis. We found that apoptotic cell numbers were dramatically reduced in lung tissues of NLRX1^−/−^ mice compared to that in WT mice (Fig. 3A,B). To ascertain the impact of NLRX1 gene deletion on apoptotic signaling, we determined mRNA levels for pro-apoptotic members of the B cell lymphoma (Bcl)-2 family, *Bax* and *Bak* (Fig. 3C,D). Activation of *Bax* and release of cytochrome *c*, which are essential for initiating the apoptotic cascade, were also confirmed by western blotting (Fig. 3E–G). These results suggest that signaling related to apoptosis declined due to the loss of NLRX1.

**Figure 4:**
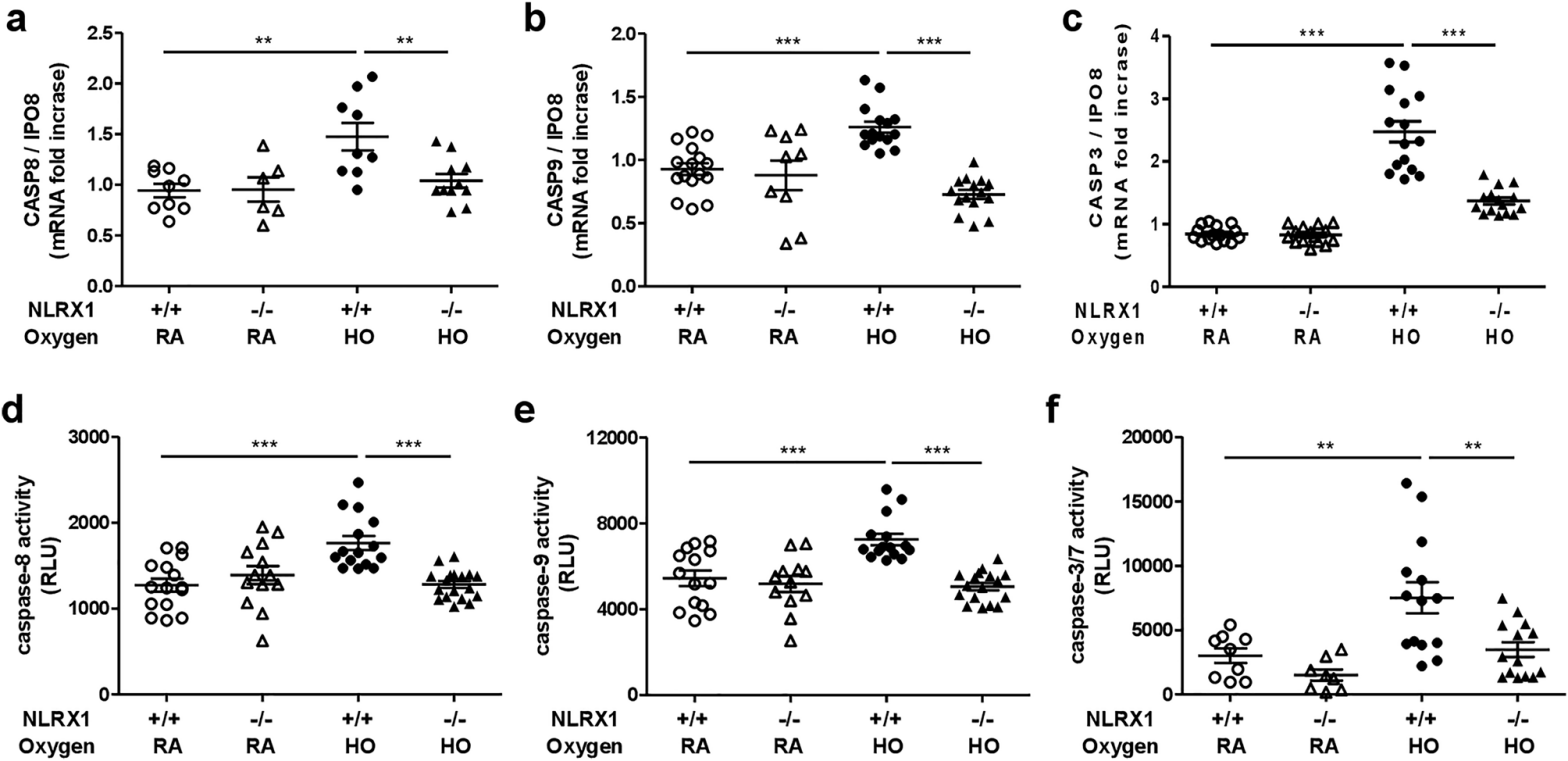
NLRX1 deficiency reduces the expression of caspase and caspase activity in hyperoxic acute lung injury. (**A**–**C**) The mRNA levels of Caspase (CASP) -8, -9, and -3 were evaluated by real-time PCR. (**D**–**F**) Caspase -8, -9, and -3/7 activities were analyzed by luminescence using protein from lung lysates. Results are presented as the mean ± SEM and are representative of at least three independent experiments (n = 5 mice per group). **p < 0.01, ***p < 0.001 (Student’s *t* test).

To further elucidate the role of NLRX1 in apoptosis, the caspase cascade was assessed using real-time PCR and caspase activity assay. We selected the representative and pivotal components of the apoptosis pathway, initiator caspase-8 and -9, and effector caspase-3 and -7. In our hyperoxic model, caspase expression and activity were significantly augmented in wild-type mice. However, hyperoxia-treated NLRX1^−/−^ mice showed reduced expression and activity of these caspase proteins tested as compared to hyperoxia-treated WT mice (Fig. 4A–F). Thus, NLRX1 deficiency inhibits apoptosis besides reducing inflammation and improving survival.

### NLRX1 deficiency suppresses MAPK signaling pathways under hyperoxic conditions

**Figure 5:**
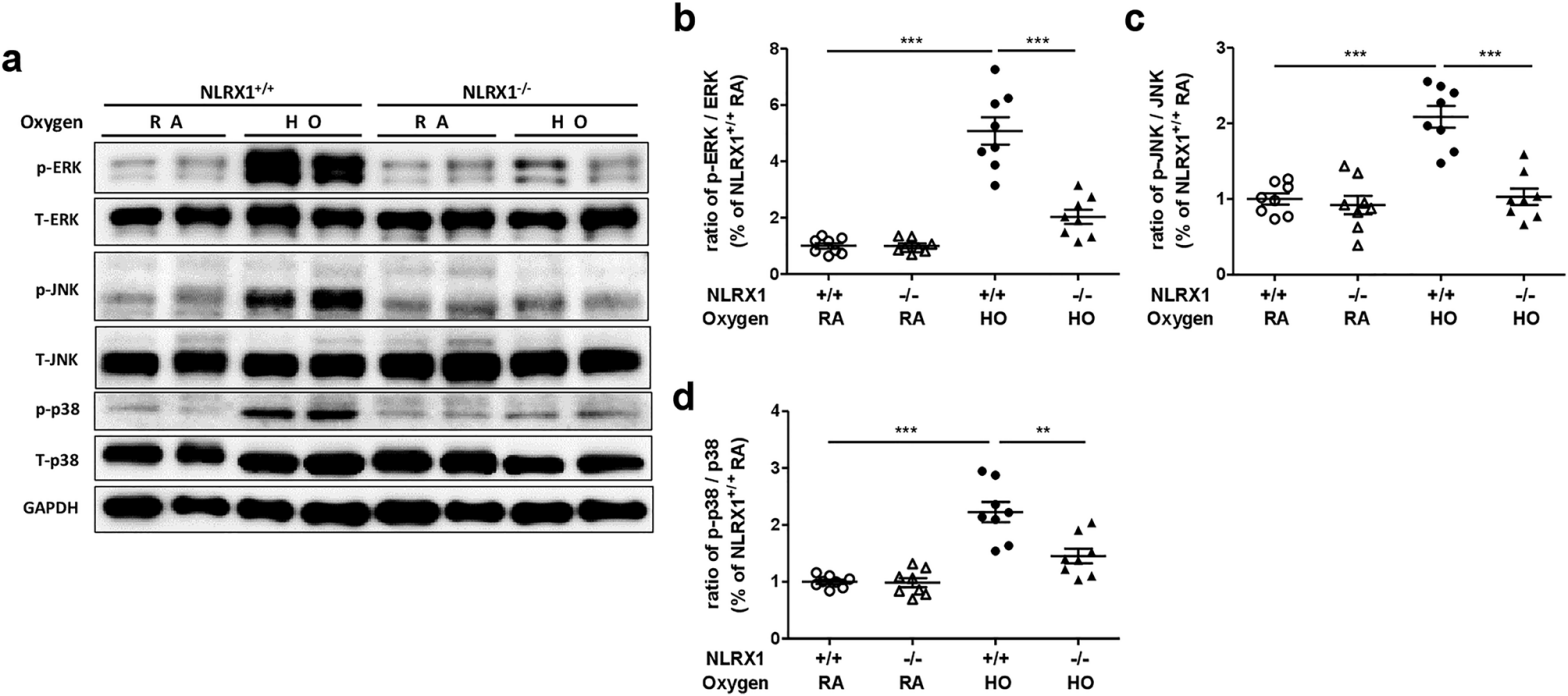
NLRX1 regulates MAPK signaling pathways in hyperoxia. (**A**) Phosphorylation of MAPK pathways (ERK 1/2, JNK, and p38) was measured by western blot analysis using lung lysates, and (**B**–**D**) the ratios of molecule signal intensity were quantified. Results are presented as the mean ± SEM and are representative of four separate experiments (n = 8 mice per group). **p < 0.01, ***p < 0.001 (Student’s *t* test).

Phosphorylation of MAPK signaling pathways is known to be modulated by hyperoxia, and NLRX1 is also known to regulate the MAPK signaling pathways. Thus, we investigated whether MAPK pathways were involved in NLRX1 function in hyperoxic lung injury. We evaluated the phosphorylation levels of ERK 1/2, JNK, and p38 in lung lysates through western blotting. As expected, hyperoxia increased phosphorylation levels in MAPK pathways in wild-type mice. However, NLRX1 deficiency attenuated the increase in MAPK pathway phosphorylation in response to hyperoxia (Fig. 5A–D).

## Discussion

In this study, we found that NLRX1 knockout alleviates HALI, particularly by inhibiting induction of apoptosis. Recent studies have suggested that NLRX1 plays a protective role in diseases, including cancer, COPD, inflammatory bowel disease, and ischemia–reperfusion injury^26^. A recent review concluded that NLRX1 inhibits the MAVS/RLH pathway and modulates the innate immune responses and the CD4^+^ T cells through immunometabolism^26^. On the contrary, other studies, including our experimental hyperoxic model, found that the absence of NLRX1 also had protective effects. In one study, deficient of NLRX1 enhanced host defense against vesicular stomatitis virus by means of increased IFN-I production and reduced autophagy^11^. NLRX1 silencing was also shown to decrease intracellular ROS generation and apoptosis involving JNK signaling in HEI-OC1 cells treated with cisplatin^8^. NLRX1 deficiency has been shown to elevate fatty acid metabolism to prevent diet-induced hepatic steatosis^28^. In cochlear hair cells, NLRX1 was essential for hair cell maturity and hearing and was also associated with age-associated increases in apoptosis through the JNK pathway^16^.

In earlier studies, NLRX1 was shown to be associated with various pulmonary diseases, such as influenza A virus infection, invasive pulmonary aspergillosis, and COPD, as well as with aging^26,29,30^. However, there have been no previous investigations of NLRX1 and HALI, even though NLRX1 is known to modulate ROS production, inflammatory leukocyte infiltration, cell death, and MAPK pathways, all of which are implicated in the pathogenesis of HALI^27,31^. In the present work, we confirmed that HALI is associated with increased NLRX1 expression (Supplementary Information).

Hyperoxia is known to affect alveolar and lung structures, interfere with lung development, and cause fibrosis, increasing the permeability of the lungs and exacerbating alveolar protein leakage^32^. This outcome was evident in the current study, where total protein concentrations were elevated in BAL fluid in mice exposed to hyperoxia. Hyperoxic treatment also increased cellular cytotoxicity, with cell death leading to increased inflammatory cell (macrophage, neutrophil, lymphocyte) infiltration and production of proinflammatory cytokines (IL-1β, IL-6, TNF-α and CCL2). Despite the prolonged exposure to a high concentration of oxygen, inflammatory responses and pathological changes were lower in NLRX1-deficient mice. Thus, NLRX1 may regulate hyperoxia-induced lung injury. A number of studies have been carried out using NLRX1-deficient macrophages, dendritic cells, and airway epithelial cells in lungs^11,29,33^, and it has been shown that NLRX1 is expressed prominently in alveolar macrophages of mice and humans^15^. Therefore, we expect that NLRX1 deficiency in macrophages might ameliorate HALI. However, further studies are needed for macrophages as the effects of regulation by NLRX1 may vary depending on experimental conditions, the stimuli applied, and the origin of the macrophages^24^.

Cell death, specifically apoptosis and necrosis, has been shown to be involved in the pathogenesis of HALI^34,35^. Apoptosis results in the accumulation of cellular and biochemical debris, which causes further injuries in lungs, thus contributing to hyperoxia toxicity^5^. The Bcl-2 family is known to regulate apoptosis, whereby pro-apoptotic members of the family, such as *Bax, Bak, Bid*, and *Bim,* induce apoptosis by increasing mitochondrial outer membrane permeability, which induces cytoplasmic leakage of pro-apoptotic agents and release cytochrome *c*^5,34,36^. When cytochrome *c* interacts with apoptotic protease activating factor (Apaf)-1, it activates the caspase cascade^37^. In caspase cascade apoptosis, activated initiator caspases (caspase 2, 8, 9, and 10) initiate apoptosis signals, and in response, effector caspases (caspase 3, 6, and 7) are activated to execute apoptosis^38^. In our study, we showed that the levels of various elements in apoptosis pathways, from the Bcl-2 family to caspase cascade proteins, were increased in response to hyperoxia. Our data showed that apoptosis induced by hyperoxia was reduced in NLRX1-deficient mice as compared to wild-type mice; the same result was found for mortality. In previous research, activated *Bax* and *Bak* were identified as key effectors in hyperoxia-induced alveolar epithelial cell apoptosis, with *Bax-* and *Bak*-deficient mice having significantly lower levels of lung injury and increased long-term survival under hyperoxic conditions^39^. In the present study, NLRX1-deficient mice showed dramatically reduced *Bax* and *Bak* expression and improved survival (*p* = 0.0007) as compared to WT mice. These results suggest that NLRX1 promotes alveolar epithelial cell apoptosis and induces lung tissue damage against oxygen toxicity and lethality via pathological apoptosis, suggesting that NLRX1 plays a critical role in the pathogenesis of hyperoxia. Pyroptosis, which is being actively studied, has many similarities to apoptosis and necrosis. However, pyroptosis is an inflammatory programmed cell death that causes the cell membrane to lose integrity and release cytokines such as IL-1β and IL-18^40–42^. Mainly, pyroptosis is induced by caspase-1-dependent canonical pathway, but recent studies have found that the inflammatory caspases 4, 5, and 11 and the apoptosis-related caspases 3 and 8 also activate the NLRP3 inflammasome to induce pyroptosis^40,43^. In the current study, we found that pyroptosis-related caspases (caspases 3 and 8) and cytokine IL-1β were reduced in NLRX1^−/−^ mice under hyperoxic conditions; hence, we speculate that NLRX1 deletion might alleviate pyroptosis under hyperoxia.

MAPK signaling pathways participate in a variety of biological processes and regulate pro- and anti-apoptotic processes^18^. The key members of MAPK pathways are ERK 1/2, p38, and JNK, which modulate cell growth, proliferation, stress responses, and survival in hyperoxia^17^. In addition, NLRX1 is known to modulate MAPK signaling^24,44^. In an LC3-associated phagocytosis model induced by *Histoplasma capsulatum*, NLRX1 deficiency attenuated MAPK signaling^25^. In the current study, we similarly found that phosphorylation of ERK 1/2, p38, and JNK was reduced in NLRX1^−/−^ mice as compared to WT mice, confirming that NLRX1 acts via the MAPK signaling pathway in regulating apoptosis under hyperoxic conditions.

One limitation of the current study is the use of a mouse model that is not entirely parallel to human disease, owing to differences in the broader physiology of mice and humans. Lung injuries induced by hyperoxia in the mouse model associate with a variety of symptoms, but cannot be equated to human lung diseases such as COPD, bronchopulmonary dysplasia (BPD), or ARDS.

In conclusion, our current study reveals that NLRX1 deficiency relieves pulmonary hyperoxic acute injuries, such as inflammation and apoptosis, and ultimately mortality, through the MAPK pathway. These investigations suggest that NLRX1 might be a valuable therapeutic target for HALI treatment.

## Methods

### Mice

Mice (C57BL/6; 6–8 weeks old, males) were purchased from Orient Bio Inc. (Seongnam, South Korea). NLRX1 knock-out (NLRX1^−/−^) mice were kindly provided by Dr. J. P. Ting (University of North Carolina). All animals used were sex-and age-matched, of the same genetic background, and housed under a 12 h dark–light cycle under specific pathogen-free (SPF) conditions and had free access to water and food during the research. Animal experiments were approved by the Institutional Animal Care & Use Committee (IACUC) of the Yonsei University (protocol No. 2021-0178; Seoul, Korea), and the study was conducted in compliance with the ARRIVE guidelines. All methods were performed in accordance with the relevant guidelines and regulations.

### Oxygen exposure

WT (NLRX1^+/+^) and NLRX1 knock-out (NLRX1^−/−^) mice were exposed to > 95% oxygen (Hyperoxia, HO) in cages enclosed in an airtight Plexiglas chamber (57 × 42 × 37 cm, JEUNG DO BIO & PLANT Co., Seoul, Korea). The chamber was maintained at atmospheric pressure. Oxygen levels were monitored for the duration of the experiment (72 h) using an oxygen analyzer (MaxO_2_^+^A, MAXTEC, Salt Lake City, UT). As controls, sex- and age-matched WT and NLRX1^−/−^ mice were housed in similar conditions under normoxia (room air, RA). For the four experimental groups (WT RA, WT HO, NLRX1^−/−^ RA, NLRX1^−/−^ HO), each group consisted of 3–5 mice, and the experiment was performed at least 3 times.

### Bronchoalveolar lavage (BAL) fluid collection and analysis

During sacrifice under anesthesia, mice were subjected to blunt dissection of the trachea. A small-caliber tube was inserted into the airway; 0.9 ml of phosphate-buffered saline (PBS) was injected into the lungs. The fluid was collected, and the collection process was repeated to obtain 1.8 ml BAL fluid. The collected BAL fluid was centrifuged at 3000 rpm for 5 min at 4 °C to separate the cell pellet and supernatant. The cell pellet was resuspended in PBS and mixed with an equal volume of Trypan Blue Solution, and the cells were then counted using a hemocytometer. BAL cells were centrifuged onto slides using a Cytospin centrifuge (Thermo Fisher Scientific, Waltham, MA). Cell differentiation was assessed using a Diff-Quik stain kit (Medion Diagnostics, Fribourg, Switzerland). The supernatant was stored at − 80 °C and used for various protein analyses and assessment of LDH activity.

### Histology and Immunohistochemistry (IHC)

The left lobes of sacrificed mice lungs were fixed in 4% formalin for 3 days and embedded in paraffin. Lung tissues were cut into 5-µm sections and stained using hematoxylin and eosin (H&E) to analyze airway inflammation. For immunohistochemical studies, sections were deparaffinized by washing twice with xylene for 10 min followed by treatment with 100% (5 min × 2), 95% (5 min), and 70% (5 min) ethanol. The tissues were then heated with retrieval buffer (DAKO, A/S, Glostrup, Denmark) for 20 min in a steamer and allowed to cool to room temperature (RT; 18–22 °C) over 20 min. After washing the sections, they were placed in peroxidase blocking solution (DAKO) for 5 min, and then in protein block solution (DAKO) for 1 h. The NLRX1 antibody (Proteintech, Rosemont, IL) or normal rabbit IgG (Santa Cruz Biotechnology, Inc, Dallas, TX) were prepared as 1:500 dilutions and applied to tissue sections, followed by incubation at 4 °C overnight. The color was developed with 3,3′-diaminobenzidine solution (DAKO), and the reaction was stopped using deionized water; the immunostained tissue sections were mounted on slides with an aqueous-base mounting medium.

### Bicinchoninic acid (BCA) assay

Protein leakage in BAL was evaluated using a Pierce™ BCA Protein Assay Kit (Thermo Fisher scientifics, Waltham, MA) according to the manufacturer’s instructions. The BAL fluid supernatant and standards were dispensed into a 96-well plate in 25 µl aliquots, and 200 µl working reagent was dispensed into each well. The plate was placed in an incubator at 37 °C for 30 min. The protein concentration in BAL fluid was measured via absorbance at 562 nm using a microplate reader (Molecular Devices, San Jose, CA).

### Lactate dehydrogenase (LDH) assay

LDH concentration in BAL fluid was measured using a cytotoxicity detection kit (Roche Applied Science, Mannheim, Germany) according to the manufacturer’s instructions. BAL fluid sample (100 µl) and 100 µl of kit reagent were mixed in a 96-well plate and allowed to react at RT for 30 min. After the reaction was completed, the reaction was stopped by adding 50 µl of stop solution, and the LDH activity was determined via absorbance at 492/690 nm using a microplate reader.

### Real-time PCR

Mouse lung tissues were homogenized using a T10 Basic Ultra-Turrax homogenizer (IKA Labortechnik, Staufen, Germany) and lysed in Trizol reagent (Invitrogen, Carlsbad, CA) according to the manufacturer’s instructions. Relative mRNA expression levels were measured by reverse transcription and real-time PCR; 1 µg of total RNA was synthesized to cDNA by reverse-transcription with a ReverTra Ace qPCR RT Master Mix Kit (Toyobo, Osaka, Japan). Real-time PCR was performed in 20 µl reactions containing 10 µl Power SYBR Green^®^ PCR Master Mix (Applied Biosystems, Waltham, MA), 1 µl cDNA template, 1 µl forward primer, 1 µl reverse primer, and deionized water to bring to the desired volume. Quantitative PCR was performed with the StepOnePlus™ Real-Time PCR System (Applied Biosystems, Waltham, MA) according to the manufacturer’s protocol. Primer pairs for real-time PCR were manufactured by MBiotech (Hanam, South Korea). The levels of mRNA were normalized to IPO8. Fold changes were calculated using the 2^− Δ ΔCT^ method.

### ELISA

The levels of interleukin-6 (IL-6) and C–C motif chemokine ligand 2 (CCL2) were quantified in BAL fluid by ELISA (R&D systems, Minneapolis, MN, USA) as per manufacturer’s instructions; 96-well plates were each coated with anti-mouse cytokine antibodies overnight at RT, and the wells were then blocked with PBS containing 1% bovine serum albumin for 1 h. Dilution standards and samples were then added to the wells and incubated for 2 h. After incubation with anti-mouse-cytokine antibodies for 2 h, followed by washing, streptavidin–horseradish peroxidase was added for 20 min, followed by washing and addition of 3,3′,5,5′-tetramethylbenzidine substrate solution (KPL) for a further 20 min. The reaction was stopped using 2 N sulfuric acid, and the plates were read at 450 nm using a microplate reader.

### Western blotting

Lung tissues were gently homogenized and lysed in RIPA lysis and extraction buffer (Thermo Fisher). The protein concentrations were measured using the Bradford assay. Equal amounts of protein samples were loaded onto 8–12% SDS-PAGE gels and separated by electrophoresis and then transferred to polyvinylidene fluoride membranes (Millipore, Bedford, MA). Membranes were blocked in Tris buffered saline containing 0.1% Tween 20 (TBST) with 5% skim milk and incubated overnight at 4 °C with primary antibodies. After washing with TBST, the membranes were incubated at room temperature with secondary antibodies for 1 h. The protein signal was analyzed using Image J software. Protein samples were normalized to GAPDH. The primary antibodies were used as follows: anti-NLRX1 (Proteintech, Rosemont, IL), anti-BAX, anti-Cyto C, anti-P-ERK 1/2, anti-T-ERK 1/2, anti-P-JNK, anti-T-JNK, anti-P-p38, anti-T-p38, and anti-GAPDH (Cell Signaling Technology, Danvers, MA).

### TUNEL assay

TUNEL assays were carried out using an in-situ Cell Death Detection Kit (AP) (Roche Applied Science, Mannheim, Germany) according to the manufacturer’s protocol. Images were obtained using a light microscope at 400× magnification in five random fields for each section and used to calculate the rates of TUNEL-positive cells.

### Caspase activity

Total protein was extracted from the lungs using RIPA lysis buffer solution and a tissue homogenizer according to the manufacturer’s protocol. In a 96-well plate, 25 µl of Caspase-Glo^®^ 3/7 Reagent (Promega, Madison, WI) and 25 µg of 1 mg/ml protein were added in a 1:1 ratio and incubation was performed for 30 min and luminescence was recorded at 30 min intervals from 30 min to 3 h. Caspase-Glo^®^ 8 Assay and Caspase-Glo^®^ 9 Assay protocols were conducted using the same methods as Caspase-Glo^®^ 3/7 Assay.

### Statistics

For the animal studies, values were expressed as means ± SEM. Most results were evaluated using Student’s *t*-test. Cell count results were compared using two-way ANOVA, and survival analysis was corrected for multiple comparisons by the log-rank test. All evaluations were performed using GraphPad Prism (GraphPad Software, Inc., San Diego, CA, USA). In all analyses, *p* < 0.05 was considered statistically significant.

## Supplementary Information

Supplementary Information.

## Data Availability

The datasets used and/or analyzed during the current study available from the corresponding author on reasonable request.
