# Supplementary material for: NLRX1 knockdown attenuates pro-apoptotic signaling and cell death in pulmonary hyperoxic acute injury
Source: Sci Rep. 2023 Mar 1;13:3441. doi: 10.1038/s41598-023-28206-x (PMC9975446; doi:10.1038/s41598-023-28206-x)

# NLRX1 knockdown attenuates pro-apoptotic signaling and cell death in pulmonary hyperoxic acute injury

**Hye Rin Kim[1], Mi Na Kim[1], Eun Gyul Kim[1], Ji Su Leem[1], Seung Min Baek[1], Yu Jin Lee[1], Kyung Won Kim[1], Min-Jong Kang[2], Tae Won Song[3*], Myung Hyun Sohn[1*]**

[1]Department of Pediatrics, Severance Hospital, Institute of Allergy, Institute for Immunology and Immunological Diseases, Severance Biomedical Science Institute, Graduate School of Medical Science, Brain Korea 21 Project, Yonsei University College of Medicine, 50-1 Yonsei-ro, Seodaemun-gu, Seoul 03722, Korea.

[2]Section of Rheumatology, Allergy and Immunology, Department of Internal Medicine, Yale University School of Medicine, New Haven, CT, USA.

[3]Department of Pediatrics, Ilsan Paik Hospital, Inje University College of Medicine, 170 Juhwa-ro, Ilsanseo-gu, Goyang 10380, Korea.

# Figure. 1. b _ NLRX1

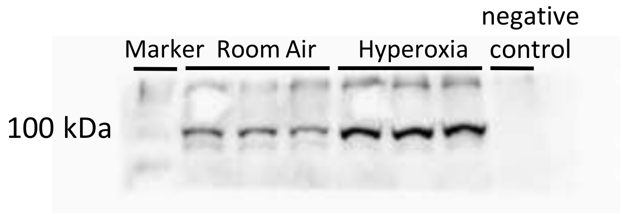


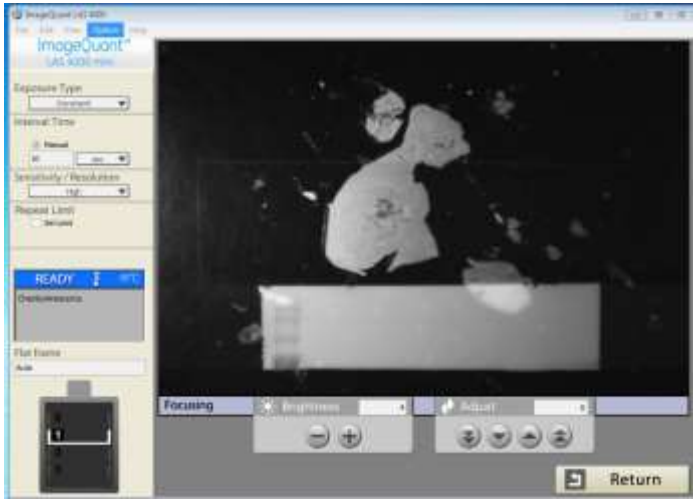


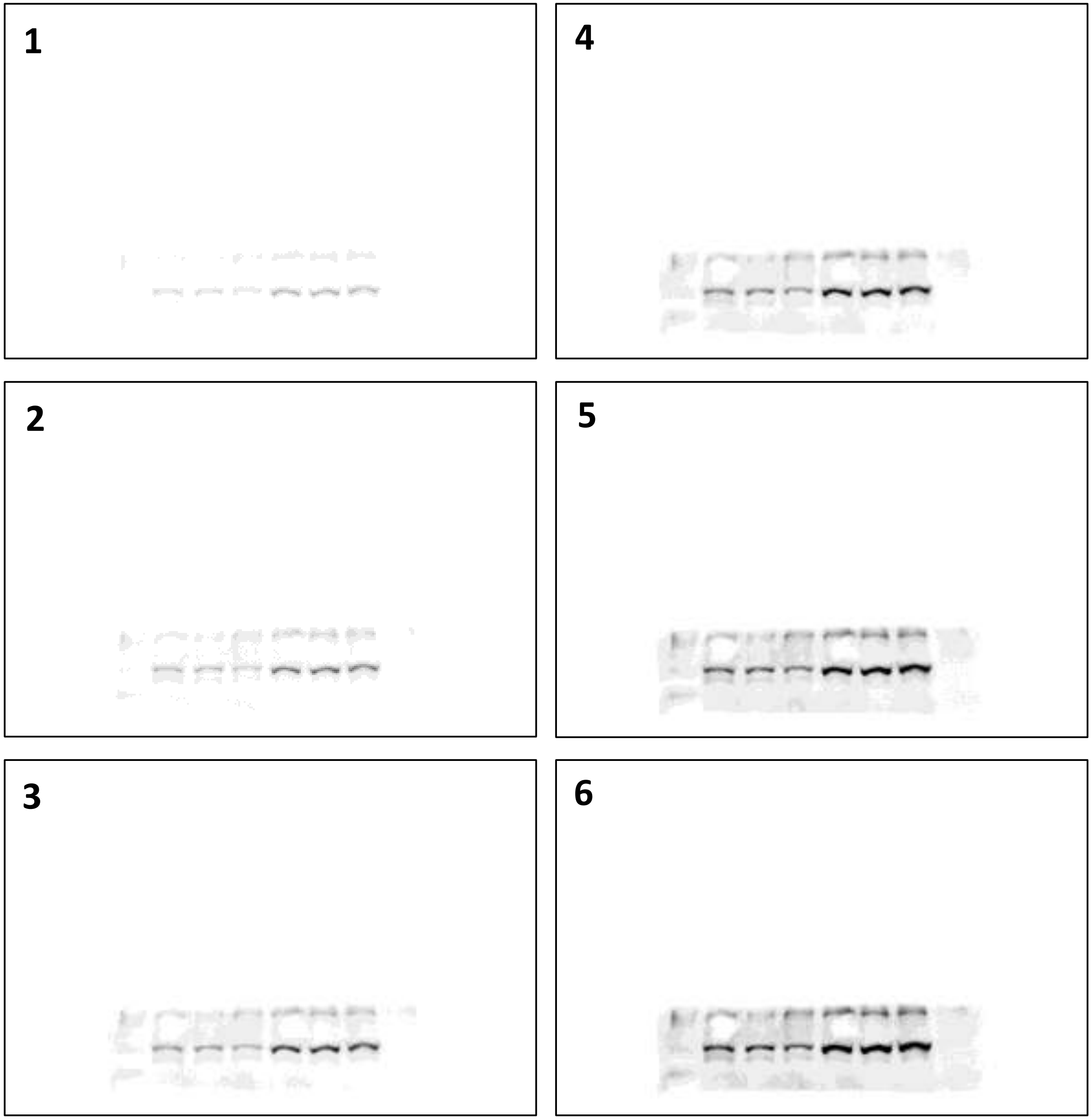


The image on the lower left is a screen capture of the equipment software and demonstrates that the membrane was cut prior to antibody hybridization. The images on the right are the original, unprocessed images taken under a series of exposure times.

Figure. 1. b _ GAPDH

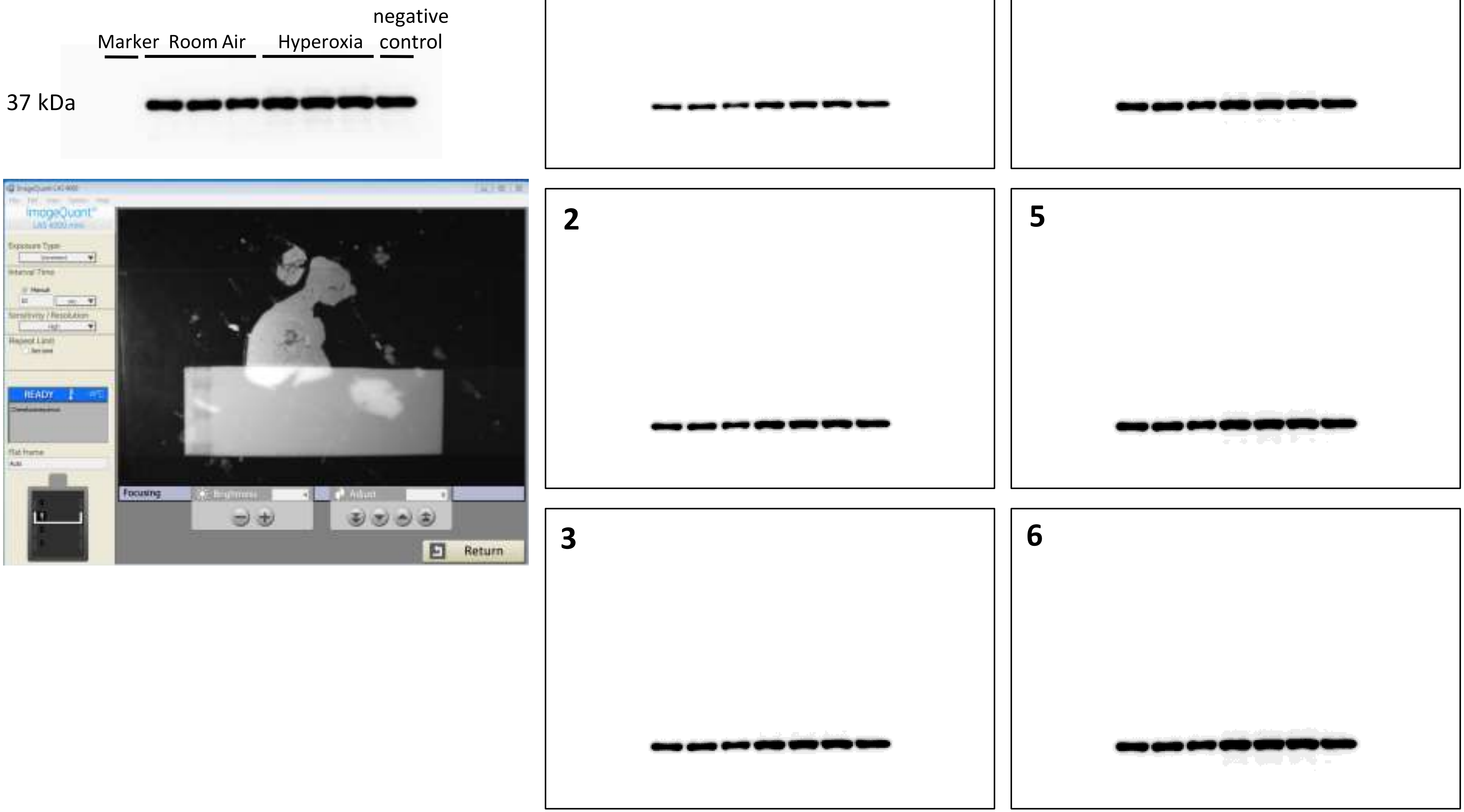


The image on the lower left is a screen capture of the equipment software and demonstrates that the membrane was cut prior to antibody hybridization. The images on the right are the original, unprocessed images taken under a series of exposure times.

Figure. 3. e _ BAX & Cytochrome C

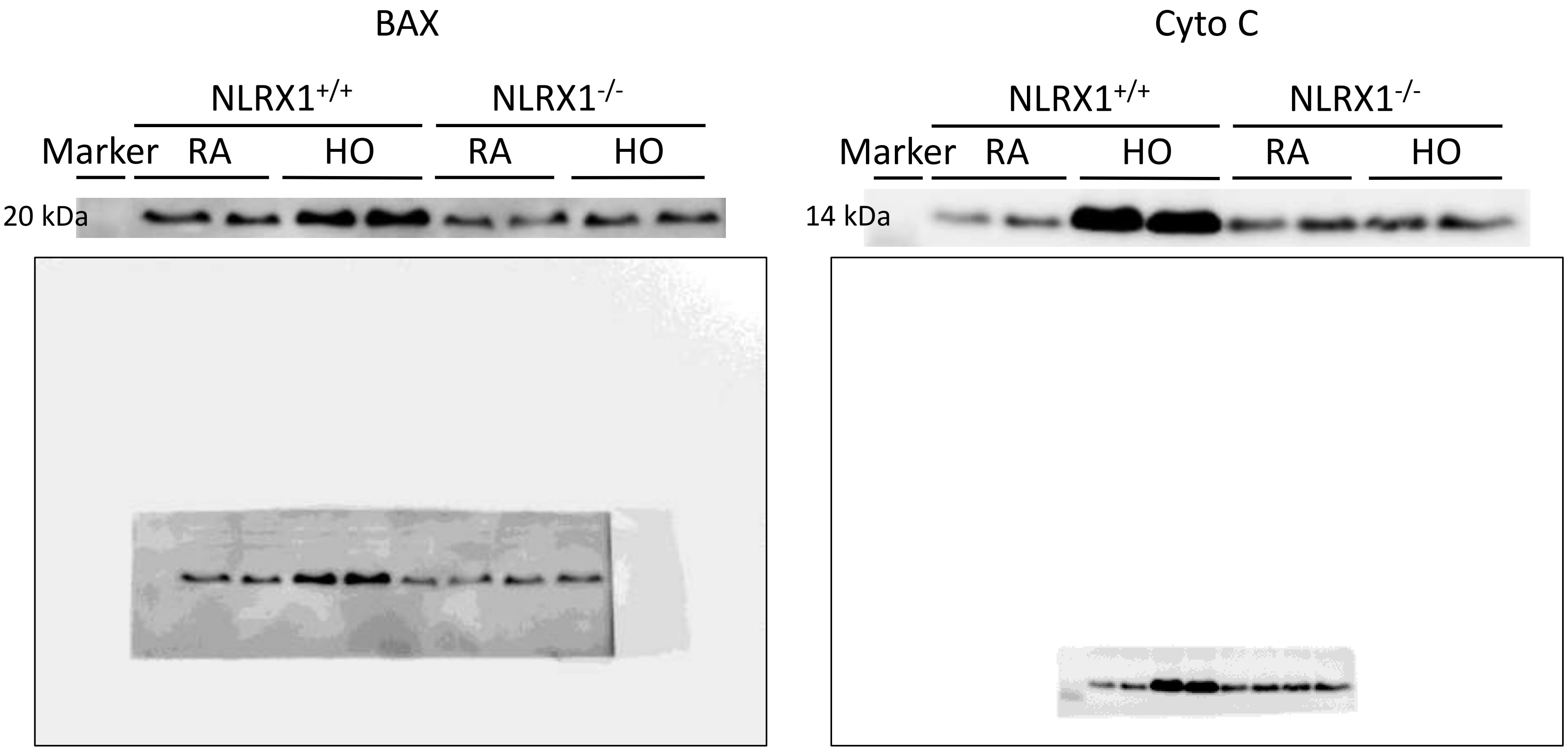


These images are the original, unprocessed images, but the membrane were cut prior to antibody hybridization. In addition, the size of the visible membrane varies according to the adjustment of the shooting distance, but all are original full-length blots.

Figure. 3. e _ GAPDH

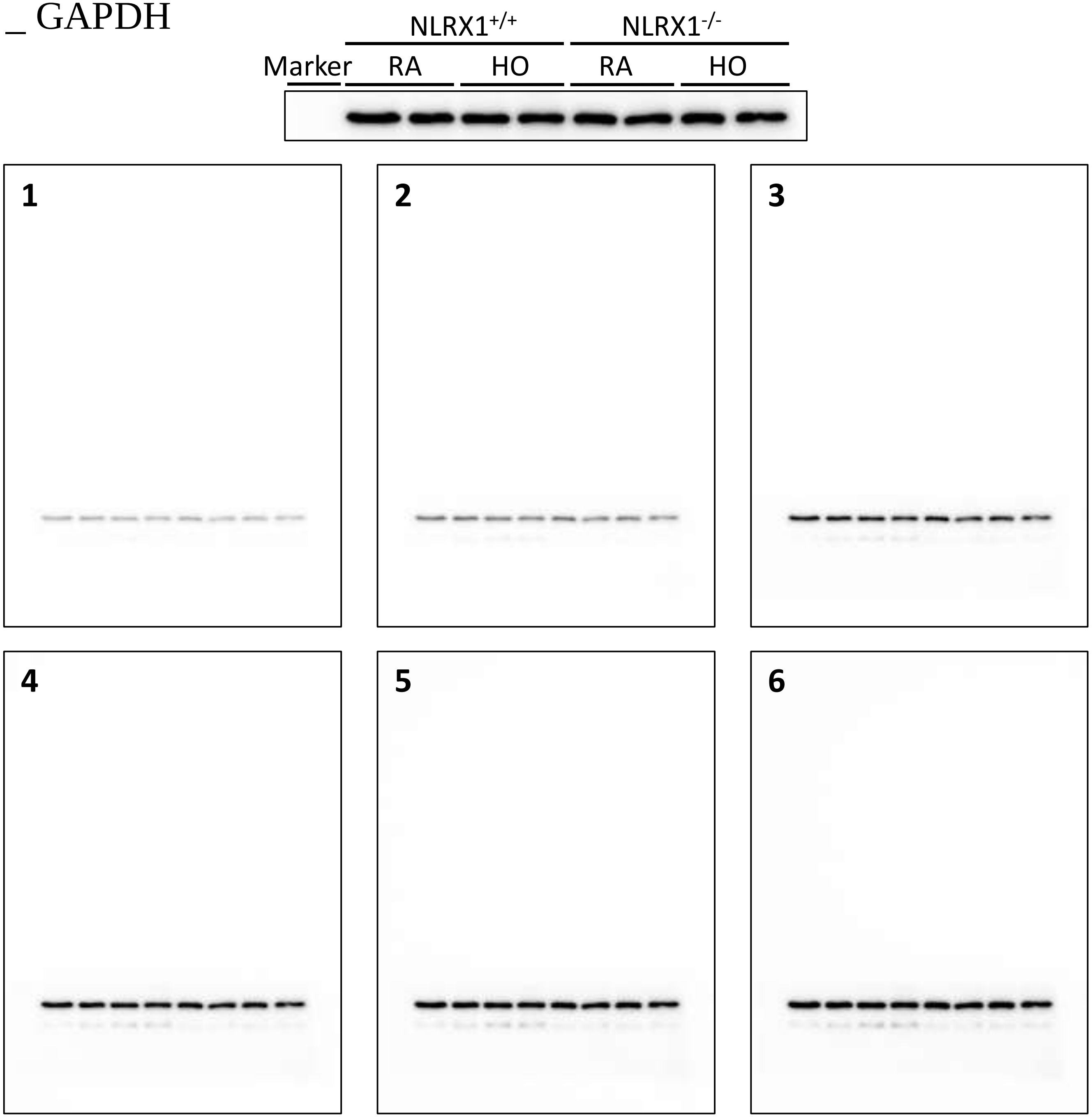

Figure. 5. a

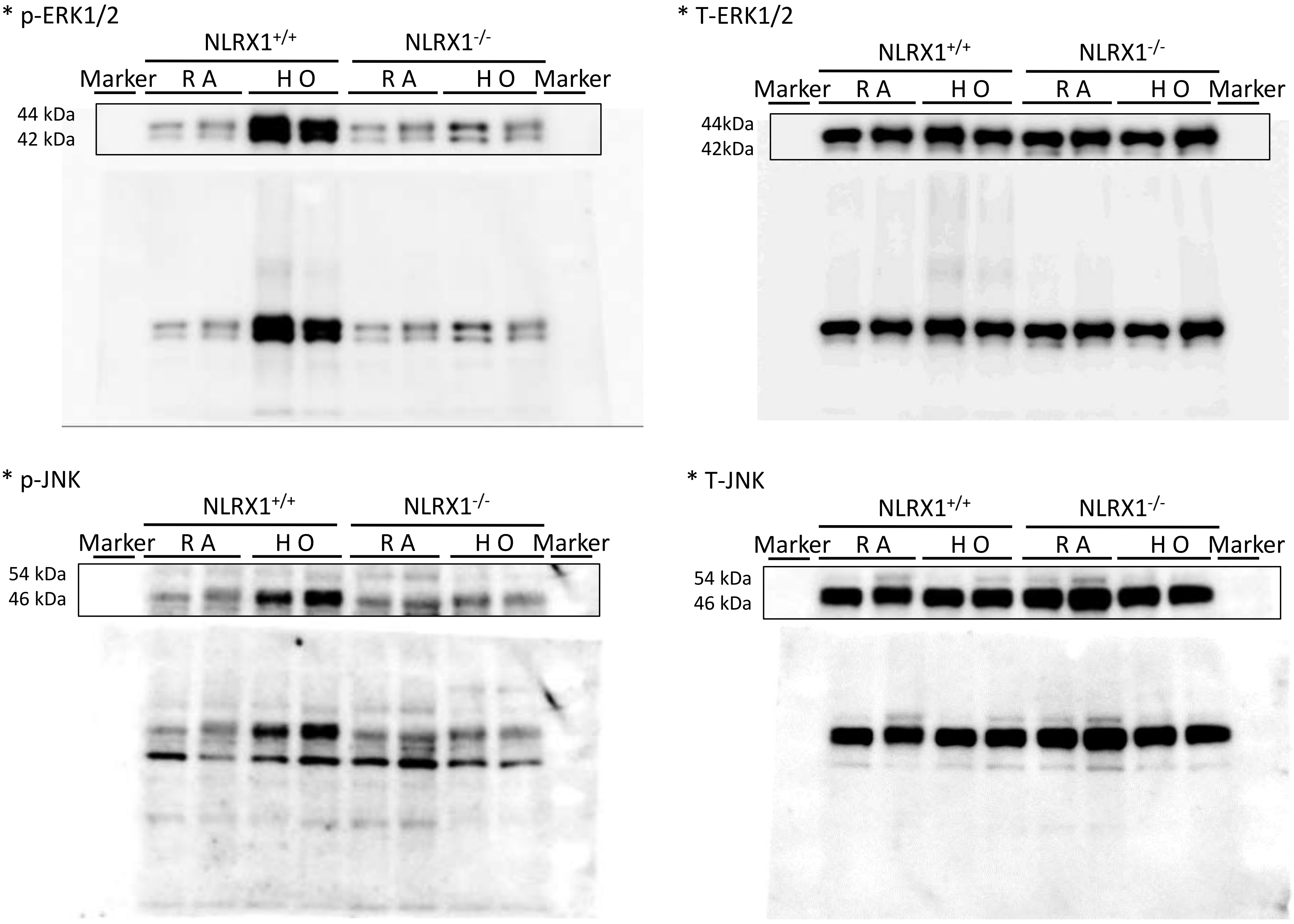
* p-ERK1/2
NLRX1+/+
NLRX1-/-
Marker
R A
H O
R A
H O
Marker
44 kDa
42 kDa
* T-ERK1/2
NLRX1+/+
NLRX1-/-
Marker
R A
H O
R A
H O
Marker
44kDa
42kDa
* p-JNK
NLRX1+/+
NLRX1-/-
Marker
R A
H O
R A
H O
Marker
54 kDa
46 kDa
* T-JNK
NLRX1+/+
NLRX1-/-
Marker
R A
H O
R A
H O
Marker
54 kDa
46 kDa

# Figure. 5. a

* p-p38

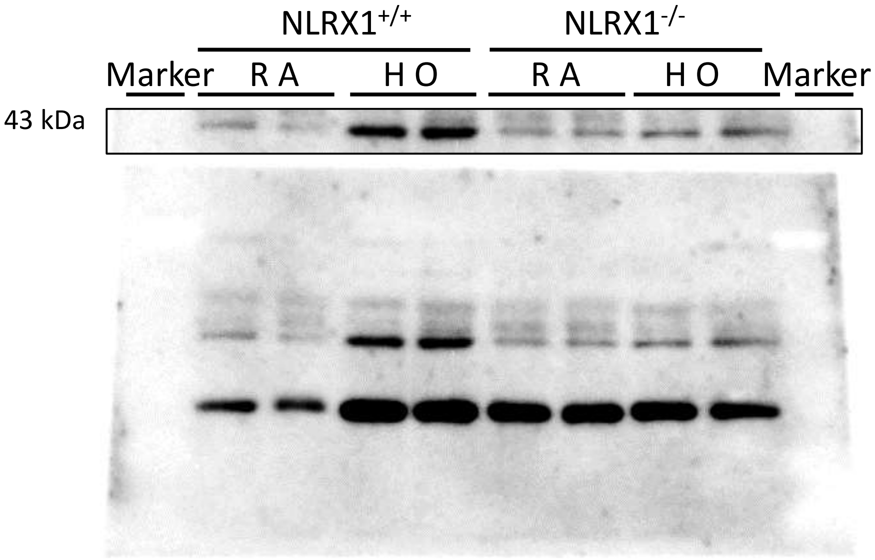


* T-p38

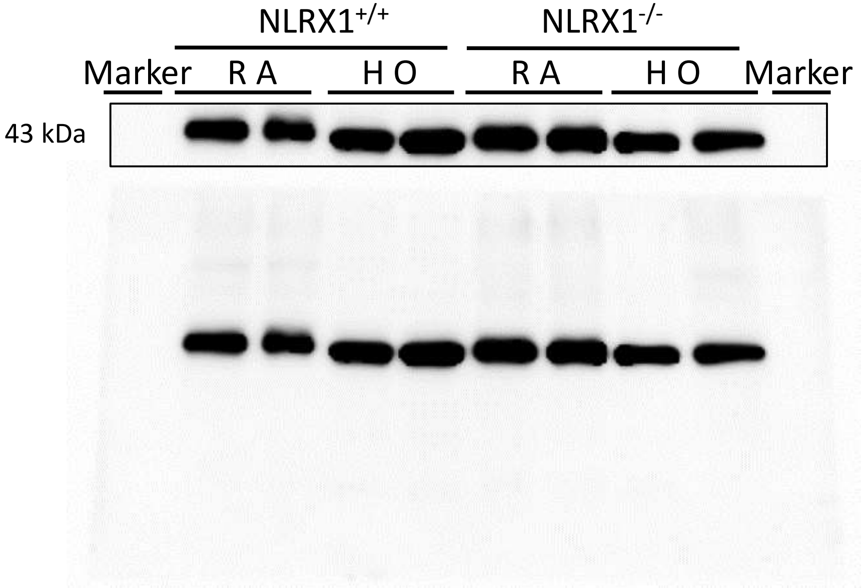


* GAPDH

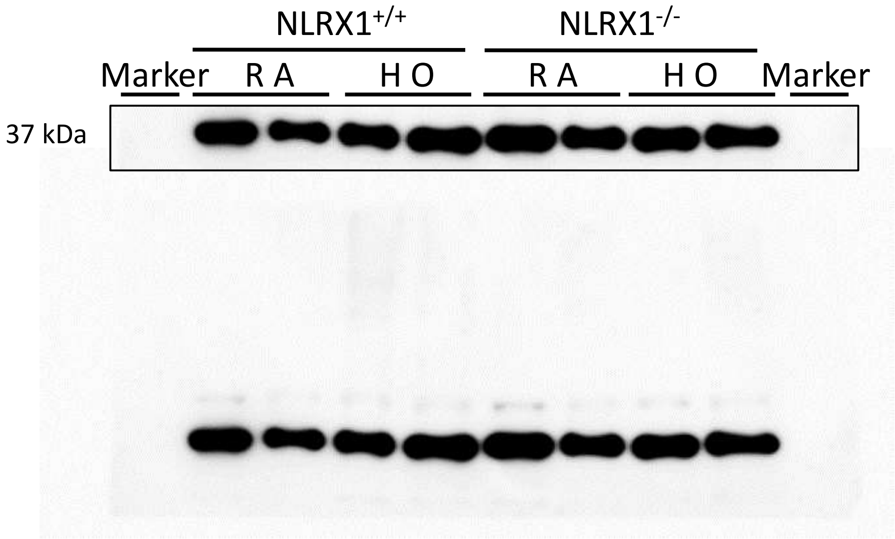

Supplement: Supplementary file 1 — Supplementary Information. [file 41598_2023_28206_MOESM1_ESM.pdf]
